# The Impact of Different Sources of Zinc, Manganese, and Copper on Broiler Performance and Excreta Output

**DOI:** 10.3390/ani12091067

**Published:** 2022-04-20

**Authors:** Steven Bryan Franklin, Marion Belinda Young, Mariana Ciacciariello

**Affiliations:** Department of Animal and Poultry Science, University of KwaZulu-Natal, Private Bag X01, Scottsville 3201, South Africa; stevenbryanfranklin@gmail.com (S.B.F.); marionbyoung@gmail.com (M.B.Y.)

**Keywords:** broiler, trace mineral, source, copper, manganese, zinc

## Abstract

**Simple Summary:**

Maximising broiler production without negatively affecting the environment is currently paramount. This study was conducted because minerals such as zinc, manganese, and copper are usually provided in excess of the animal’s requirements, which leads to excretion and potential environmental contamination. Different sources of these minerals were tested to determine the best dietary source that would optimize broiler chicken growth whilst reducing the excretion of excess minerals to the environment. This study showed that these minerals provided in their hydroxy form reduced the excretion to the environment without affecting the growth, feed intake, or feed-to-gain ratio of broiler chickens.

**Abstract:**

Commercial premixes provide trace minerals (TM) such as zinc (Zn), manganese (Mn), and copper (Cu) in excess of the requirements to maximize broiler performance. High inclusion levels of TM in broiler feed and their low absorption in the gastrointestinal tract leads to increased levels of TM in the excreta, resulting in the contamination of the environment. A 35-day broiler trial was conducted with 2880 one-day-old Cobb broiler males to test the effect of the supplementation of different sources of TM on growth performance, while evaluating levels in the excreta. Inorganic (ITM), organic (OTM), and hydroxy (HTM) sources of TM were tested against a positive control of current recommended levels of ITM. At 35 d, birds fed HTM were 55 g (*p* < 0.05) heavier than those fed ITM at the same inclusion level. In contrast, birds fed the control, OTM, and HTM showed no significant difference in body weight. Providing broilers with HTM significantly (*p* < 0.05) reduced Zn and Cu excretion at 35 d of when compared to those who were fed diets containing ITM or PC. Supplementing different sources of TM to broiler diets at levels below the recommendations showed no negative effect on broiler performance. The use of HTM significantly reduced TM excretion in broilers. The use of HTM in broiler diets can maintain broiler performance and reduce the negative impact on the environment.

## 1. Introduction

In commercial practice, broiler premixes provide trace minerals (TM) such as zinc (Zn), manganese (Mn), and copper (Cu) in excess to the birds’ requirements to maximize broiler performance and to provide a safety margin due to the heterogeneity of raw materials [1,2]. The Cobb 500 Broiler Performance and Nutrition Supplement [3] recommends that Zn, Mn, and Cu be supplemented at 100, 100, and 15 mg/kg, respectively. The National Research Council (NRC) [4] recommends 40 mg Zn/kg, 60 mg Mn/kg, and 8 mg Cu/kg feed. Cobb 500 recommendations, therefore, provide approximately twice the amount of TM recommended by the NRC.

The relationship between mineral sources and the ability to meet the requirements of the broilers is poorly understood. Thus, TM have been included in broiler diets at levels higher than required, and due to low absorption of TM in the gastrointestinal tract (GIT) of the broiler, there is a resultant increase of TM in the broiler excreta [5,6]. In recent years, this has caused growing concern because excess accumulation of TM in the soil and water can have a detrimental effect on plants, microorganisms, livestock, and humans [7,8,9]. Therefore, it is important to find ways to reduce TM excretion without affecting the growth performance of the birds and increasing production costs.

The use of inorganic TM (ITM) at lower levels provides a cost-effective way of optimizing animal health and production. However, trace mineral salts could dissolve in the low pH of the upper GIT, reducing absorption and bioavailability [10] due to nutrient and ingredient antagonisms [11]. Organic TM (OTM) have been suggested as a possible solution to this problem. Due to the higher bioavailability, sources such as proteinates and amino acid chelates have been used in premixes [12]. Replacing commercial levels of inorganic Zn, Mn, and Cu with lower levels of organic Zn, Mn, and Cu did not compromise broiler performance and significantly reduced TM levels in the litter [5,13]. The implication is that OTM can be included in diets at a much lower concentration than ITM without negatively impacting production performance, whilst lowering TM excretion [12]. An alternative source is hydroxy chloride TM (HTM), which the EU recently approved as a feed additive for all animal species [14]. However, data are limited on the effects of HTM on TM excretion. The unique crystalline structure is highly stable, unlike ITM and OTM, leading to improved feed stability and reducing the loss of other nutrients such as vitamins and enzymes [15].

The objective of this study was to investigate the effect of inorganic, organic, and hydroxy Zn, Mn, and Cu on broiler growth performance. A second objective was to evaluate which Zn, Mn, and Cu sources produced the least TM in broiler excreta after 35 days.

## 2. Materials and Methods

### 2.1. Birds and Housing

Two thousand eight hundred and eighty day-old Cobb 500 broiler males were fed to 35 d of age. Sixty birds were allocated to forty eight floor pens in an environmentally controlled house, with a stocking density of 17.6 birds/m^2^. Birds were randomly allocated to four dietary treatments with twelve replications each. Chicks, from a commercial hatchery, were vent sexed and vaccinated for Newcastle disease and infectious bursal disease before arrival. Water and feed were provided ad libitum for the duration of the trial. Each pen contained two water fonts and two pan feeders for the first 7 days. Two tube feeders per pen and water nipple lines were used thereafter, as per commercial recommendations [3]. Wood shavings were used as bedding material, and gas spot-brooders provided heat to the birds. The temperature was regulated by thermostat control. The initial temperature was set at 31.5 °C for the first 2 days and decreased by 1.5 °C daily until day 22, after which the temperature remained at 21.5 °C for the duration of the trial. All chicks received 24 h of light on the first day, 23L:1D from days 1 to 6, and 16L:8D from days 7 to 35. The trial was approved by the Animal Ethics Committee of the University of KwaZulu Natal (reference number AREC/022/016).

### 2.2. Experimental Treatments

The feeding schedule comprised a starter crumble (0–12 days), a pelleted grower (13–24 days), and a pelleted finisher feed (25–35 days) (Table 1). The four treatments consisted of a standard maize–soyabean, commercial ration (Pietermaritzburg, South Africa) with the vitamin and mineral premixes differing in TM content per treatment. The treatments were as follows: Treatment 1 (positive control, PC): Cobb-Vantress Inc. (Siloam Springs, AR, USA) levels of Zn, Mn, and Cu [3] using ITM; Treatment 2: NRC levels of Zn, Mn, or Cu [4] using ITM; Treatment 3: NRC levels of Zn, Mn, and Cu using OTM; and Treatment 4: NRC levels of Zn, Mn, and Cu using HTM. Supplementary levels of TM are shown in Table 2.

### 2.3. Performance Parameters

All chicks were weighed as a collective group per pen, and the average individual weight per bird at 0, 7, 14, 21, 28, and 35 d was calculated. These values were used to ascertain the birds’ body weight (BW) and average daily gain (ADG). Cumulative feed intake (FI) per pen was calculated as the difference between the amount of feed given and the amount of feed remaining in the feeders on days 7, 14, 21, 28, and 35. Mortalities and culls were recorded daily to calculate FI and feed conversion ratio (FCR; feed-to-gain ratio) accurately.

### 2.4. Mineral Excretion Collection and Analysis

On day 35, one bird that represented the pen population was stunned and euthanized by cervical dislocation (12 birds per treatment). The portion of the large intestine containing excreta from the vent to the caeca was dissected, weighed, and stored in a freezer at −20 °C until further analysis. The excreta samples were dried at 60 °C overnight, and then ground until caking was observed. Dry ashing of 2 g of ground excreta at 550 °C for 4 h was performed [16]. A mixture of hydrochloric and nitric acid was used to dissolve the ashes (AOAC Official Method 985.01). The Inductively Coupled Plasma method (AOAC Official Method 2011.14) was then used to determine Zn, Mn, and Cu concentrations in the excreta samples (Vista MPX, Varian Inc., Palo Alto, CA, USA).

### 2.5. Experimental Design and Statistical Analysis

Two thousand, eight hundred and eighty chicks were allocated to forty-eight pens with sixty chicks per pen. The forty-eight pens were divided into four treatments with twelve replications per treatment. A randomized complete blocks design was used to compare the response in BW, FI, FCR, mortality, and Zn, Mn and Cu levels in excreta to different sources of TM in the diet, with the pen location serving as a blocking factor. Data were analysed using one-way analysis of variance (JMP^®^, Version 15, SAS Institute Inc., Cary, NC, USA, 1989–2021). Tukey’s multiple range test was used to determine differences between treatment means at a significance level of *p* < 0.05.

## 3. Results

### 3.1. Broiler Growth Performance

The effects of different sources of TM on broiler performance from 0 to 35 d are displayed in Table 3. Inorganic, organic, and hydroxy sources of Zn, Mn, and Cu did not affect broiler growth performance from 0 to 21 d (*p* < 0.05). At 28 d, those birds supplemented with HTM were 40 g heavier (*p* < 0.05) than those fed ITM at the same inclusion level. Birds fed HTM outperformed those fed ITM at 28 d, with birds gaining, on average, 1.5 g more per day (*p* < 0.05). The broilers fed ITM, OTM, and HTM treatments did not differ in BW, ADG, FI, or FCR at 28 d of age from those fed the PC. Birds supplemented with HTM were 55 g heavier (*p* < 0.05) and gained, on average, 1.6 g more (*p* < 0.05) than the ITM treatment at 35 d. No significant difference in broiler BW or ADG was observed when comparing the PC to the ITM, OTM, and HTM treatments at 35 d. At the conclusion of the trial, there were no treatment differences in FI or FCR.

### 3.2. Trace Mineral Excretion

**Figure 1:**
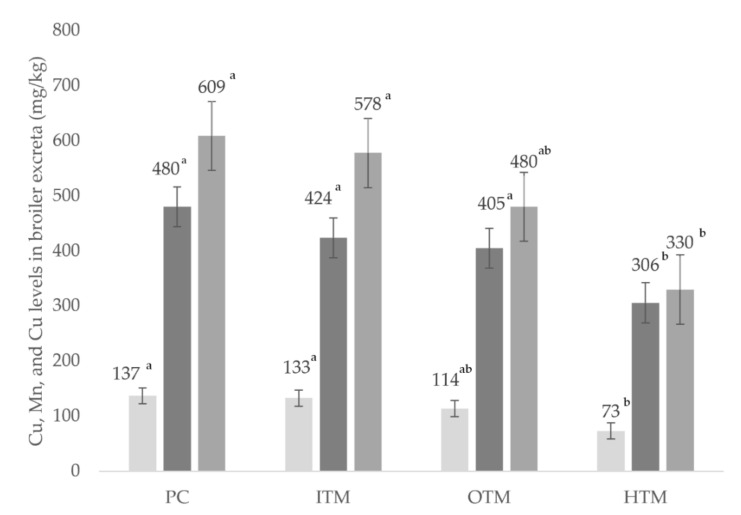
Zinc, manganese, and copper broiler excreta output at 35 days with different levels of supplementation in the feed. PC, positive control; ITM, inorganic trace minerals; OTM, organic trace minerals; HTM, hydroxy trace minerals; ^a,b^ minerals in different treatments with different superscripts differ significantly at *p* < 0.05. *n* = 24.

The fecal excretory output of TM at 35 days is shown in Figure 1. Providing broilers with HTM reduced (*p* < 0.05) Zn and Cu excretion at 35 d of age compared to those diets containing ITM or PC. There were no significant differences in the Zn and Cu excretion between the OTM- and HTM-supplemented diets. A 174 mg/kg (*p* < 0.05) difference was recorded between the PC and HTM diets; however, there were no significant differences amongst the remaining treatments.

## 4. Discussion

This study aimed to determine whether supplementing broilers with different sources of TM over a 35-day period would affect broiler growth performance. The literature reported to date presents the study of two sources of TM at a time, or evaluates much higher inclusion levels of TM, especially older studies. The current work presents a comparison with three generation sources of TM and has been conducted mimicking commercial conditions, in contrast with the available research.

The findings showed that from 0 to 21 d, BW, ADG, FI, and FCR were not significantly affected by different sources of TM. Luo et al. [17] observed similar findings in a 21 d broiler performance trial using HTM and ITM. Birds supplemented with 150 mg/kg or 300 mg/kg inorganic Cu showed no significant differences in BW, ADG, FI, or FCR compared to those supplemented with tribasic Cu chloride at the same levels. This study showed that even at ITM levels twice that of HTM, no differences in growth performance were observed.

Similarly, Miles et al. [18] showed that broiler diets supplemented with 150 mg Cu/kg from inorganic and hydroxy sources had no significant effect on broiler BW or FI at 21 d of age. The response to OTM at 21 d in the current experiment was similar to Star et al. [19]. Performance parameters such as BW gain, FI, and FCR of birds at 21 d of age did not differ between organic and inorganic sources of Zn, Mn, and Cu at equal and graded supplemental levels. Likewise, Gheisari et al. [12] studied the effects of lowering OTM levels fed to broilers compared to those fed ITM and showed that birds fed organic sources of Zn, Mn, and Cu did not outperform those fed inorganic sources in terms of BW, FI, and FCR at 21 d.

In the current study from 28 d onwards, broilers fed HTM outperformed (*p* < 0.05) those fed ITM at the same supplementary levels of Zn, Mn, and Cu in BW and ADG. There were no significant differences in BW or ADG of birds fed the PC or OTM compared to those fed HTM, indicating that the source type could have improved the performance of birds fed HTM supplements. However, the literature is limited on growth performance results in broilers supplemented with HTM at 28 d. Contrasting results on the use of OTM use can be found in the literature on broiler performance at 28 d. Nollet et al. [13] concluded that BW, FI, and FCR were not significantly affected in broilers supplemented with OTM compared to ITM at 28 d; however, El-Husseiny et al. [20] suggested that BW and FCR could be improved using OTM at half the commercial level of ITM.

At 35 d, broilers fed HTM again outperformed those fed the ITM treatment in BW and ADG, but the use of ITM at commercial levels (PC) did not significantly improve broiler growth performance. Limited research has been conducted that compares standard levels of ITM to HTM on broiler growth performance at 35 d. Conly et al. [21] showed contrasting results that when using supplemental inorganic and hydroxy Mn (at equal levels) of 3600 and 4500 mg/kg, no significant differences were exhibited in BW and FI. Broiler growth performance also varies in the literature when supplementing broilers with equal and different supplemental levels of ITM and OTM. Graded and equal levels of organic Cu up to 250 mg/kg did not improve or reduce the BW or FCR performance of broilers grown to 35 d compared to those fed inorganic sources [22]. However, the use of organic Cu at 500 mg/kg increased BW but maintained FCR compared to inorganic Cu. In the present study, results disagreed with those found by El Husseiny et al. [20], who found that birds fed diets with OTM had higher BW, ate less feed, and showed improved FCR when compared to those birds on ITM-supplemented diets.

The results of the current study up to 21 d suggest that the dietary requirements for TM were met, whether supplemented with ITM, OTM, or HTM, and any additional TM present had no significant effect on growth performance. Therefore, it is possible to use HTM and OTM at NRC at recommended levels, instead of ITM at Cobb standards, and maintain broiler growth parameters. Despite being an older source of TM, the effects observed in the OTM treatments could be due to the positive effects that diets with OTM have on the development of the GIT [23]

The second aim of this study was to determine which source of Zn, Mn and Cu would produce the least amount of TM in broiler excreta. Broiler diets supplemented with OTM instead of ITM did not reduce Cu, Mn, and Zn excretion. Previous research by Dozier et al. [24] indicated similar responses to the above experiment when Zn was supplemented at the same levels using either inorganic or organic sources. No difference in TM excreta levels was observed between OTM and ITM. Burrel et al. [25] concurred with the above results where organic Zn supplemented to birds did not reduce the amount of Zn in the excreta compared to those fed inorganic Zn. Conversely, Nollet et al. [13] found that replacing ITM with lower levels of OTM resulted in fecal amounts of Zn, Mn, and Cu being significantly reduced. Manangi et al. [5] suggested that broiler chickens supplemented with organic sources of Zn, Mn, and Cu produced less TM in the excreta than birds fed inorganic sources of Zn, Mn, and Cu. Research on the use of HTM to reduce TM in excreta was not found to corroborate the results of this study. Further research is needed to reduce the excretion of TM in poultry manure.

## 5. Conclusions

This study indicated that supplementation of ITM, OTM, and HTM at NRC levels could match the performance of broilers supplemented with commercial Cobb levels of TM. Compared to ITM, the supplementation with HTM at NRC levels resulted in improved BW and ADG and reduced Zn and Cu in the excreta. The experiment suggests that using HTM at lower levels than ITM can maintain broiler performance while reducing the amount of TM excreted in the feces. Further trials at a commercial level should determine whether the use of HTM would be an economically viable and an environmentally sustainable method of supplementing broilers with Zn, Mn, and Cu. Reduced supplementary levels of ITM, as well as no supplementation, can sustain broiler performance. Decreased ITM has the added benefit of a lower TM excretion. The use of HTM, despite being a costlier form of TM, could result in a positive impact on the environment while maintaining growth performance.

## Figures and Tables

**Table 1:** The composition and calculated nutrient content of broiler rations (as-fed) in the starter (0–12 days), grower (13–24 days) and finisher (25–35 days) phases.

Ingredient		Starter (0–12 Days)		Grower (13–24 Days)		Finisher (25–35 Days)	
		*	#	*	#	*	#
Yellow Maize	%	53.7	53.5	56.6	56.5	58.5	58.3
Soya Oilcake (46%)	%	33.9	33.9	31.6	31.6	29.9	30.0
Sunflower Oilcake	%	4.0	4.0	4.0	4.0	4.0	4.0
Sunflower Oil	%	4.92	4.98	4.97	5.03	5.16	5.22
Limestone	%	1.47	1.46	1.21	1.21	1.06	1.06
MCP	%	0.53	0.53	0.23	0.23		
Salt	%	0.48	0.48	0.41	0.41	0.41	0.41
DL Methionine	%	0.28	0.28	0.26	0.26	0.27	0.27
HCL Lysine	%	0.26	0.26	0.26	0.26	0.25	0.25
Threonine	%	0.07	0.07	0.06	0.06	0.06	0.06
Choline Chloride	%	0.08	0.08	0.08	0.08	0.08	0.08
Vitamin and Mineral Premix	%	0.30	0.40	0.30	0.40	0.30	0.40
Total		100	100	100	100	100	100
Nutrient Composition (Analysed)							
Moisture	%	11.1	11.1	11.2	11.2	11.4	11.4
Protein	%	21.5	21.5	20.6	20.6	19.9	19.9
Total Lysine	%	1.33	1.33	1.27	1.27	1.22	1.22
Total Methionine	%	0.61	0.61	0.58	0.58	0.58	0.58
Energy	MJ/kg	12.2	12.2	12.4	12.4	12.6	12.6
Fat	%	6.68	6.74	6.78	6.83	7.15	7.20
Starch	%	35.3	35.2	37.0	36.9	37.7	37.6
Fibre	%	3.80	3.79	3.80	3.80	3.62	3.62
Ash	%	5.69	5.68	4.95	4.95	4.44	4.44
Calcium	%	0.74	0.74	0.59	0.59	0.49	0.49
Phosphorus	%	0.52	0.52	0.45	0.45	0.41	0.41
Sodium	%	0.21	0.21	0.18	0.18	0.18	0.18
Potassium	%	0.96	0.96	0.92	0.92	0.89	0.89
Chloride	%	0.40	0.40	0.36	0.36	0.36	0.36

* Composition of positive control (PC), Treatment 2 (inorganic trace minerals), and Treatment 4 (hydroxy trace minerals); # Composition of Treatment 3 (organic trace minerals). Note: Basal diets differ slightly between * and # due to vitamin and mineral inclusion levels. Ingredient composition for the premix: Vitamin A 10,000 IU, Vitamin D3 10,000 IU, Vitamin E 50 mg, Vitamin K 4 mg, Vitamin B1 5 mg, Vitamin B2 8 mg, Vitamin B6 4 mg, Vitamin B12 0.02 mg, niacin 50 mg, pantothenic acid 20 mg, folic acid 2 mg, ferrous sulphate 100 mg, selenium 0.35 mg.

**Table 2:** Sources and supplementary levels of trace minerals fed in the broiler starter (0–12 days), grower (13–24), and finisher (25–35 days) rations.

Treatment	Source of Trace Minerals	Standard	Zn	Mn (mg/kg)	Cu (mg/kg)
			(mg/kg)		
1 (PC)	inorganic	Cobb	100	100	15
2	inorganic	NRC	40	60	8
3	organic	NRC	40	60	8
4	hydroxy	NRC	40	60	8

PC, positive control; NRC, National Research Council [4].

**Table 3:** Effects of different sources of trace minerals on broiler performance from 0 to 35 days.

Treatments	*n*	BW (g)	ADG (g)	FI (g)	FCR
7 days					
PC	12	162	17.7	158	0.98
ITM	12	162	17.7	154	0.95
OTM	12	163	17.8	154	0.95
HTM	12	165	18.1	159	0.97
*p* Value		0.47	0.59	0.13	0.55
LSD		6.55	0.97	6.61	0.06
14 days					
PC	12	484	31.9	536	1.11
ITM	12	482	31.7	533	1.11
OTM	12	488	32.2	535	1.10
HTM	12	489	32.2	539	1.10
*p* Value		0.35	0.38	0.64	0.62
LSD		11.47	0.83	12.26	0.02
21 days					
PC	12	1043	47.9	1245	1.19
ITM	12	1033	47.4	1234	1.19
OTM	12	1039	47.7	1233	1.19
HTM	12	1045	48.0	1249	1.2
*p* Value		0.50	0.50	0.35	0.65
LSD		22.7	1.09	28.4	0.02
28 days					
PC	12	1770 ^ab^	61.9 ^ab^	2297	1.30
ITM	12	1747 ^a^	61.0 ^a^	2279	1.31
OTM	12	1756 ^ab^	61.4 ^ab^	2291	1.31
HTM	12	1787 ^b^	62.5 ^b^	2299	1.29
*p* Value		0.02	0.02	0.72	0.14
LSD		33.8	1.21	51.1	0.02
35 days					
PC	12	2503 ^ab^	70.4 ^ab^	3593	1.44
ITM	12	2453 ^a^	69.0 ^a^	3536	1.44
OTM	12	2487 ^ab^	70.0 ^ab^	3542	1.43
HTM	12	2508 ^b^	70.6 ^b^	3559	1.42
*p* Value		0.04	0.04	0.32	0.28
LSD		54.3	1.55	88.2	0.03

BW, body weight; ADG, average daily gain; FI, cumulative feed intake; FCR, feed conversion ratio (feed:gain); PC, positive control; ITM, inorganic trace minerals; OTM, organic trace minerals; HTM, hydroxy trace minerals; LSD, least significant difference; ^a,b^ means with different superscripts within a column are significantly different at *p* < 0.05.

## Data Availability

Data are available from the corresponding author upon request.
